# Supplementary material for: The lipid raft marker flotillin FloA drives relocalization of the plasma membrane H+-ATPase PmaA as a protective response to calcium stress
Source: PLoS Genet. 2026 Oct 29;22(10):e1012147. doi: 10.1371/journal.pgen.1012147 (PMC13734606; doi:10.1371/journal.pgen.1012147)
Supplement: Supplementary file 1 [file pgen.1012147.s001.pdf]

S1 Table. Strains used in this study

Organism	Strain	Genotype	Source
A. nidulans	TN02A3	*pyrG89, argB2; pyroA4, nkuA::argB*	[1]
	BPU1	*pyrG89, biA1, wA3; pyroA4*	[2]
	SJW02	*wA3; argB::trpCΔB; pyroA4;* $alcA_p$*-GFP-tubA*	[3]
	RMS012	*biA1/+, +/pabaA1, +/yA2; argB::trpCΔB/argB::trpCΔB; methG1/+; veA1/veA1, trpC801/trpC801*	[4]
	R21	*pabaA4, yA1*	FGSC
	floA-GFP	*pyrG89, biA1, wA3, floA-sfGFP,* $pyr4^{Ncra}$*; pyroA4*	This study
	$alcA_p$*-floA-GFP*	*pyrG89, biA1, wA3; pyroA4; argB;* $alcA_p$*-floA-sfGFP,* $pyroA^{Afum}$	This study
	$alcA_p$*-floA-3×HA-miniTurboID*	*pyrG89, biA1, wA3; pyroA4;* $alcA_p$*-floA-3×HA-miniTurboID,* $pyroA^{Afum}$	This study
	$alcA_p$*-3×HA-miniTurboID*	*pyrG89, biA,; wA3; pyroA4::* $alcA_p$*-3×HA-miniTurboID,* $pyroA^{Afum}$	This study
	floA-nLuc	*pyrG89, biA1, wA3, floA-nLuc,* $pyr4^{Ncra}$*; pyroA4;*	This study
	floA-nLuc; ΔcrzA	*pyrG89, biA1, wA3, floA-nLuc,* $pyr4^{Ncra}$*; pyroA4; crzA::*$pyroA^{Afum}$	This study

Continued on next page

Organism	Strain	Genotype	Source
A. nidulans	*floA-nLuc; ΔsltA*	*pyrG89, biA1, wA3, floA-nLuc, $pyr4^{Ncra}$; pyroA4; sltA::$pyroA^{Afum}$*	This study
	floA-nLuc; Δmac1	*pyrG89, biA1; wA3, floA-nLuc, $pyr4^{Ncra}$; pyroA4; mac1::$pyroA^{Afum}$*	This study
	floA-nLuc; ΔcnaA	*pyrG89, biA1, wA3, floA-nLuc, $pyr4^{Ncra}$; pyroA4; cnaA::$pyroA^{Afum}$*	This study
	pmaA-mCherry	*pyrG89; argB2, pmaA-mCherry; $pyrG^{Afum}$; pyroA4, nkuA::argB;*	This study
	$alcA_p$-floA-GFP; pmaA-mCherry	*pyrG89, biA1, wA3; argB, pmaA-mCherry; $pyrG^{Afum}$; pyroA4; $alcA_p$-floA-sfGFP, $pyroA^{Afum}$*	This study
	floA/ΔfloA	*biA1/+, +/pabaA1, +/yA2, floA/floA::argB; argB::trpCΔB/argB::trpCΔB; methG1/+; veA1/veA1, trpC801/trpC801;*	This study
	ΔfloA	*pabaA1, yA2, floA::argB; argB::trpCΔB; trpC801*	This study
	ΔfloA; pmaA-mCherry	*pabaA1, yA2, floA::argB; argB::trpCΔB; pmaA-mCherry, $pyrG^{Afum}$; trpC801*	This study
	$floA^c$; pmaA-mCherry	*pabaA1, yA2; pmaA-mCherry, $pyrG^{Afum}$* (*ΔfloA; pmaA-mCherry* crossed with TN02A3)	This study
A. fumigatus	A1160$^+$	*$akuB^{ku80}$::$pyrG^-$-zeo; $pyrG^-$::pyrG; MAT1-1*	[5]
	ΔAfcrzA	*akuBku80:: $pyrG^-$-zeo; ; $pyrG^-$::pyrG; MAT1-1; AFUB_007280::hph*	[6]
