# Supplementary material for: The lipid raft marker flotillin FloA drives relocalization of the plasma membrane H+-ATPase PmaA as a protective response to calcium stress
Source: PLoS Genet. 2026 Oct 29;22(10):e1012147. doi: 10.1371/journal.pgen.1012147 (PMC13734606; doi:10.1371/journal.pgen.1012147)
Supplement: Supplementary file 2 [file pgen.1012147.s002.pdf]

S2 Table. List of primers used in this study

Primer	Sequence (5’ -> 3’)
backbone.FOR	gtttaaaccctatagtgagtcgtattacgc
backbone.REV	gtttaaaccctttagtgagggttaattgcg
linear.FOR	tgattacgccaagcgcgcaat
linear.REV	gagcgcgcgtaatacgactc
floA.FOR	atgaggtacgtgctagcttttctg
floA.REV	cttgttagccacagccttctgt
floA_up.FOR	caacgacgcattcctcgct
floA_down.REV	gctatagaggggttcgtcagg
floA_5HOM1kb$^+$.FOR	gcgatcagtgacttaccctacagc
floA_3HOM1kb$^+$.REV	gctcagcatcatcgtttatgagatg
argB_CDS.FOR	cagttatttgtggatgccctcgttc
argB_CDS.REV	cgaaaccctccaaggcgg
MK15_5HOM.FOR	ccctcactaaagggtttaaacaacgaattttggcactacgg
MK15_5HOM.REV	gataccagggagcgaggaatgcgtcgt
MK15_pyroA.FOR	cctcgctccctggtatcatggttgttggg
MK15_pyroA.REV	tcagcttttcaattgtcttatggcgctgtcagg
MK15_alcAp.FOR	cataagacaattgaaaagctgattgtgatagttcccact
MK15_alcAp.REV	gtacctcattttgaggcgaggtgataggat
MK15_floA.FOR	cgcctcaaaatgaggtacgtgctagcttttctg
MK15_TicI.REV	gggtttaaacctgctaagaccgcagta
MK15_backbone.FOR	gtcttagcaggtttaaaccctatagtgagtcg
MK1_floA.FOR	ccctcactaaagggtttaaacatgaggtacgtgctagcttttctg

Continued on next page

Primer	Sequence (5’ -> 3’)
MK1_floA.REV	gctcaccatcttgttagccacagccttc
MK1_GFP.FOR	tggctaacaagatggtgagcaagggcga
MK1_GFP.REV	ccaccatctcaccatgtttcgcgcg
MK1_TicI.FOR	gaaacatggtgagatggtggatagtcaag
MK1_TicI.REV	gaagcagtatgcctgctaagaccgcag
MK1_pyr4.FOR	cttagcaggcatactgcttcctcaacc
MK1_pyr4.REV	aggtaggcggggatcttcatcattcgtcgc
MK1_3HOM.FOR	gatccccgcctacctgacgaacccct
MK1_3HOM.REV	cgactcactatagggtttaaacgcaacagggacagcaccg
MK18_HA-mTurbo.FOR	tgtggctaacaagtacccgtatgatgttccgga
MK18_HA-mTurbo.REV	accatcttacttttcggcagaccgcagac
MK18_TicI.FOR	tgccgaaaagtaagatggtggatagtcaagag
MK18_floA.REV	atacgggtacttgttagccacagccttc
MK24_nLuc.FOR	gggtggaggctctatggtcttcacactcgaagatttc
MK24_nLuc.REV	atctcacgccagaatgcgttcgc
MK24_TicI.FOR	cattctggcgtgagatggtggatagtcaa
MK24_floA.REV	catagagcctccacccttgttagccacagcct
MK44_5HOM.FOR	ccctcactaaagggtttaaactaacatagcgacctttaatctgctgc
MK44_5HOM.REV	ccatgataccagggggcaaagagctatgcagacaag
MK44_pyroA.FOR	catagctctttgccccctggtatcatggttgttggg
MK44_pyroA.REV	gattgtccaaggcaaaattgtcttatggcgctgtcagg
MK44_3HOM.FOR	cgccataagacaattttgccttggacaatctacccttc
MK44_3HOM.REV	cgactcactatagggtttaaacaccctctcatcctgcgttcc

Continued on next page

Primer	Sequence (5’ -> 3’)
MK47_5HOM.FOR	ttaaccctcactaaagggtttaaacgcggtattagtagtcgagggatca
MK47_5HOM.REV	caaccatgataccagggggtgaaagacatgaaggatgcagc
MK47_pyroA.FOR	gtctttcaccccctggtatcatggttgttggg
MK47_pyroA.REV	ccggatgaactattgtcttatggcgctgtcagg
MK47_3HOM.FOR	acagcgccataagacaatagttcatccggcacagcc
MK47_3HOM.REV	cgactcactatagggtttaaacctaagtaaaccagcaaccagaaacacg
MK50v2_5HOM.FOR	taaccctcactaaagggtttaaaccatcccattccgtctcttttcc
MK50v2_5HOM.REV	caaccatgataccagggctgtccatgaccatgtacctttgtta
MK50v2_pyroA.FOR	gtacatggtcatggacagccctggtatcatggttgttggg
MK50v2_pyroA.REV	gtacgcagggaaataattgtcttatggcgctgtcagg
MK50v2_3HOM.FOR	gcgccataagacaattatttccctgcgtacaggcaaac
MK50v2_3HOM.REV	cgactcactatagggtttaaacttagagaccaccagggccg
MK109_5HOM.FOR	ccctcactaaagggtttaaaccgagccccccaaatgcc
MK109_5HOM.REV	gataccaggggagaagcatgttctgtcgcgg
MK109_pyroA.FOR	agaacatgcttctcccctggtatcatggttgttgggtc
MK109_pyroA.REV	tctataaggggaaattgtcttatggcgctgtcaggg
MK109_3HOM.FOR	cagcgccataagacaatttccccttatagacaccatctaacacg
MK109_3HOM.REV	cgactcactatagggtttaaacgatttaaggcaagatgaacccaccc
MK26_5HOM.FOR	cctcactaaagggtttaaacctatataagtatctttccccctttc
MK26_5HOM.REV	aagcagtatgcgatggcggttctccaat
MK26_pyr4.FOR	gaaccgccatcgcatactgcttcctcaac
MK26_pyr4.REV	tcagcttttcaggggatcttcatcattc

Continued on next page

Primer	Sequence (5’ -> 3’)
MK26_alcAp.FOR	agatcccctgaaaagctgattgtgatag
MK26_alcAp.REV	catacgggtacattttgaggcgaggtgatag
MK26_mTurbo.FOR	tcgcctcaaaatgtacccgtatgatgttccg
MK26_TicI.REV	ccactcacacctgctaagaccgcagtag
MK26_3HOM.FOR	cttagcaggtgtgagtggaaatgtgtaac
MK26_3HOM.REV	tacgactcactatagggtttaaacttctagagacaatcactggac
MK61_5HOM.REV	caattattgcatgcagcgaggaatgcgtcgttg
MK61_argB.FOR	gcattcctcgctgcatgcaataattgcagcaaatattg
MK61_argB.REV	cgtcaggtaggcctcagaagaatgtgctggatagaggt
MK22_5HOM.FOR	tttaaacttgatgggaccggaacgcagc
MK22_3HOM.REV	ggtgattgtagttcgagagatctctgggc
MK61_3HOM.FOR	cattcttctgaggcctacctgacgaacccctc
MK92_5HOM.FOR	ccctcactaaagggtttaaacggtttcgccgaagttttccc
MK92_5HOM.REV	gctgaccatctcatcgtcctcaggctcagc
MK92_3HOM.FOR	gccatctaggccacgctttcgctcatcattc
MK92_3HOM.REV	cgactcactatagggtttaaactgtacgtgtcccatttac
MK92_ptrA.FOR	gcggtcttagcagaataatgcagggccctcgg
MK92_ptrA.REV	gcgaaagcgtggcctagatggcctcttgc
MK92_mCherry.FOR	gaggacgatgagatggtcagcaagggcgaag
MK92_mCherry.REV	ccaccatcctacttgtacagctcgtccatgcc
MK92_TicI.FOR	ctgtacaagtaggatggtggatagtcaagagccg
MK92_TicI.REV	cctgcattattctgctaagaccgcagtagtcc

Continued on next page

**Primer**	**Sequence (5’ -> 3’)**
Afu_floA.FOR	tcactacatggaacactggg
Afu_floA.REV	tggattgtgctgaaaagagg
Afu_actA.FOR	gggatgacatggagaagatctg
Afu_actA.REV	gcgttgaaggtctcgaagac
