# Supplementary material for: The lipid raft marker flotillin FloA drives relocalization of the plasma membrane H+-ATPase PmaA as a protective response to calcium stress
Source: PLoS Genet. 2026 Oct 29;22(10):e1012147. doi: 10.1371/journal.pgen.1012147 (PMC13734606; doi:10.1371/journal.pgen.1012147)
Supplement: Supplementary file 3 [file pgen.1012147.s003.pdf]

S1 Text. Generation of *A. nidulans* mutant strains

If not stated otherwise, DNA fragments were assembled using the NEBuilder HiFi DNA assembly master mix (New England Biolabs) according to the manufacturer's instructions. All fungal transformations were carried out as previously described [7]. Unless stated otherwise, the pUC18 backbone was amplified from plasmid pCH008 [8] (primer pair: backbone.FOR/backbone.REV) and used as the backbone for all plasmids constructed in this study. All other constructs except for pMK061 were linearized by PCR using primers linear.FOR/linear.REV, resulting in a construct excluding the vector backbone.

For tagging of FloA (AN6177) with superfolderGFP (sfGFP) at the C-terminus, the plasmid pMK001 was constructed and integrated into the genomic locus of *floA* in the BPU1 strain. Briefly, the DNA fragments covering the coding sequence of *floA* (MK1_floA.FOR/MK1_floA.REV) and the 3'- flanking region of *floA* (MK1_3HOM.FOR/MK1_3HOM.REV) were amplified from *A. nidulans* genomic DNA. The terminator region of the gene *Afu4g13510* Ticl was amplified from *A. fumigatus* genomic DNA (MK1_Ticl.FOR/MK1_Ticl.REV). the sfGFP gene (MK1_GFP.FOR/MK1_GFP.REV) was amplified from plasmid pCH008 [8]. The *Neurospora crassa pyr4* gene (MK1 _pyr4.FOR/MK1 _pyr4.REV) was amplified from plasmid pAL4 [9]. All DNA fragments were assembled in the order of *floA*, sfGFP, Ticl, *pyr4*, and *floA* 3'-flanking region and vector backbone, resulting in plasmid pMK001. After transformation of *A. nidulans* BPU1 with the plasmid, the recombinant strain *floA-GFP* was obtained.

For expression of FloA-sfGFP by the *alcA* promoter, the DNA fragment of *floA-sfGFP-Ticl* was amplified from pMK001 (MK15 floA.FOR/MK15_Ticl.REV). The DNA fragments of *alcA*$_p$ (MK15_alcAp.FOR/MK15_alcAp.REV) and the 2 kb upstream region of *floA* (MK15_5HOM.FOR/MK15_5HOM.REV) were amplified from *A. nidulans* genomic DNA. The *A. fumigatus pyroA* gene (MK15_pyroA.FOR/MK15_pyroA.REV) was amplified from genomic DNA. The pUC18 vector backbone (MK15_backbone.FOR/backbone.REV) was amplified from plasmid pCH008 [8]. All DNA fragments were assembled in the order of *floA* 5' flanking region, *pyroA*, *alcA*$_p$, *floA*, sfGFP, Ticl and vector backbone, resulting in plasmid

pMK015. This plasmid was integrated ectopically into the *A. nidulans* BPU1 strain, resulting in the recombinant strain *alcA*$_p$-*floA-GFP*.

For FloA-3×HA-miniTurboID expression by *alcA*$_p$, three repeats of the human influenza hemagglutinin (HA) epitope tag fused to miniTurboID were amplified from pLJ-HscA-mT [10]. The generated DNA fragment was then subcloned into pMK015 amplified with MK18_TicI.FOR/MK18 floA.REV, yielding pMK018. This plasmid was integrated ectopically into the *A. nidulans* BPU1 strain, resulting in the recombinant strain *alcA*$_p$-*floA-3×HA-miniTurboID*.

For 3×HA-miniTurboID expression by *alcA*$_p$, the 5'– flanking region (MK26_5HOM.FOR/MK26_5HOM.REV) and 3'-flanking region (MK26_3HOM.FOR/MK26_3HOM.REV) of *pyrG* and *alcA*$_p$ (MK26_alcAp.FOR/MK26_alcAp.REV) were amplified from *A. nidulans* genomic DNA. The *N. crassa pyr4* gene (MK26 _pyr4.FOR/MK26 _pyr4.REV) was amplified from pAL4 [9] and 3×HA-miniTurboID-Ticl (MK26_mTurbo.FOR/MK26_Ticl.REV) was amplified from pMK018. All DNA fragments were assembled in the order of 5' flanking region of *pyrG*, *pyr4*, *alcA*$_p$, 3×HA-miniTurboID, 3'-flanking region of *pyrG* and vector backbone, resulting in plasmid pMK026. This construct was linearized and used to transform the *A. nidulans* BPU1 strain, resulting in the recombinant strain *alcA*$_p$-*3×HA-miniTurboID*.

For tagging of FloA with nLuc, the human codon-optimized *nLuc* was amplified from plasmid pMM12-orsAp-nLuc-GFPs [7] with a 12 bp linker on the N-terminus (MK24_nLuc.FOR/MK24_nLuc.REV). The generated DNA fragment was subcloned into pMK001 amplified with MK24_Ticl.FOR/MK24 floA.REV, yielding pMK024. This construct was linearized and used to transform the *A. nidulans* BPU1 strain, resulting in the recombinant strain *floA-nLuc*.

For deletion of the *crzA* (AN5726) gene, the 5'– flanking region (MK44_5HOM.FOR/MK44_5HOM.REV) and 3'- flanking region (MK44_3HOM.FOR/MK44_3HOM.REV) of *crzA* were amplified from *A. nidulans* genomic DNA. *pyroA* was amplified from *A. fumigatus* genomic DNA (MK44_pyroA.FOR/MK44_pyroA.REV). The generated DNA fragments were ligated

together with the vector backbone, so that *pyroA* was located between the two flanking regions. The resulting plasmid pMK044 was linearized and used to transform the *floA-nLuc* strain, yielding *floA-nLuc;* Δ*crzA*.

For deletion of the *mac1* (AN0658) gene, the 5'-flanking region (MK47_5HOM.FOR/MK47_5HOM.REV) and 3'- flanking region (MK47_3HOM.FOR/MK47_3HOM.REV) regions of *mac1* were amplified from *A. nidulans* genomic DNA. *pyroA* was amplified from *A. fumigatus* genomic DNA (MK47_pyroA.FOR/MK47_pyroA.REV). The generated DNA fragments were ligated together with the vector backbone, so that *pyroA* was located between the two flanking regions. The resulting plasmid pMK047 was linearized and used to transform the *floA-nLuc* strain, yielding *floA-nLuc;* Δ*mac1*.

For deletion of *sltA* (AN2919), the 1 kb upstream (MK50v2_5HOM.FOR/MK50v2_5HOM.REV) and 1 kb downstream (MK50v2_3HOM.FOR/MK50v2_3HOM.REV) regions of the *sltA*-coding sequence were amplified from *A. nidulans* genomic DNA. *pyroA* was amplified from *A. fumigatus* genomic DNA (MK50v2_pyroA.FOR/MK50v2_pyroA.REV). The generated DNA fragments were ligated with the vector backbone, so that *pyroA* was located between the two flanking regions. The resulting plasmid pMK050v2 was linearized and used to transform the *floA-nLuc* strain, yielding *floA-nLuc;* Δ*sltA*.

For deletion of the *cnaA* (AN8820) gene, its 5'- flanking region (MK109_5HOM.FOR/MK109_5HOM.REV) and 3'– flanking region (MK109_3HOM.FOR/MK109_3HOM.REV) were amplified from *A. nidulans* genomic DNA. *pyroA* was amplified from *A. fumigatus* genomic DNA (MK109_pyroA.FOR/MK109_pyroA.REV). The generated DNA fragments were linked with the vector backbone, so that *pyroA* was located between the two flanking regions. The resulting plasmid pMK109 was linearized and used to transform the *floA-nLuc* strain, yielding *floA-nLuc;* Δ*cnaA*.

For deletion of *floA*, its 5'-flanking region (MK15_5HOM.FOR/MK61_5HOM.REV) and 3'- flanking region (MK61_3HOM.FOR/MK1_3HOM.REV) and the *argB* gene

(MK61_argB.FOR/MK61_argB.REV) were amplified from *A. nidulans* genomic DNA. The generated DNA fragments were linked with the vector backbone in the order of 5'-flanking region of *floA*, *argB*, 3'- flanking region of *floA* and vector backbone, resulting in plasmid pMK061. This construct was linearized (MK22_5HOM.FOR/MK22_3HOM.REV) and used to transform the *A. nidulans* diploid strain RMS012, resulting in the recombinant strain *floA/*Δ*floA*. This strain was haploidized on AMM agar plates containing 0.5 µg/mL benomyl (Merck Chemicals) yielding the Δ*floA* haploid strain.

For tagging of PmaA (AN4859) with mCherry, the 1 kb downstream region of the *pmaA*-coding sequence (MK92_5HOM.FOR/MK92_5HOM.REV) and 3'-flanking region of *pmaA* (MK92_3HOM.FOR/MK92_3HOM.REV) were amplified from *A. nidulans* genomic DNA. *ptrA* (MK92_ptrA.FOR/MK92_ptrA.REV) was amplified from pCH008 [8]. The *A. fumigatus* codon-optimized mCherry (MK92 mCherry.FOR/MK92 mCherry.REV) was amplified from pSK496 [11]. TicI (MK92_TicI.FOR/MK92_TicI.REV) was amplified from *A. fumigatus* genomic DNA. All DNA fragments were assembled on the vector backbone in the order of 1 kb downstream region of *pmaA*, mCherry, TicI and the 3'-flanking region of *pmaA*, resulting in plasmid pMK092. This construct was linearized and used to transform the *A. nidulans* strains TN02A3, *alcA*$_p$-*floA-GFP* and Δ*floA*, resulting in the recombinant strain *pmaA-mCherry, alcA*$_p$-*floA-GFP; pmaA-mCherry* and Δ*floA; pmaA-mCherry*, respectively.

For complementation of *floA* in strain Δ*floA; pmaA-mCherry*, the strain was crossed with strain TN02A3 as described previously [12, 13]. After screening by PCR, the selected progeny was named *floA*^{c}*; pmaA-mCherry*.
