# Supplementary material for: The lipid raft marker flotillin FloA drives relocalization of the plasma membrane H+-ATPase PmaA as a protective response to calcium stress
Source: PLoS Genet. 2026 Oct 29;22(10):e1012147. doi: 10.1371/journal.pgen.1012147 (PMC13734606; doi:10.1371/journal.pgen.1012147)

Raw blot image of Fig. 3a

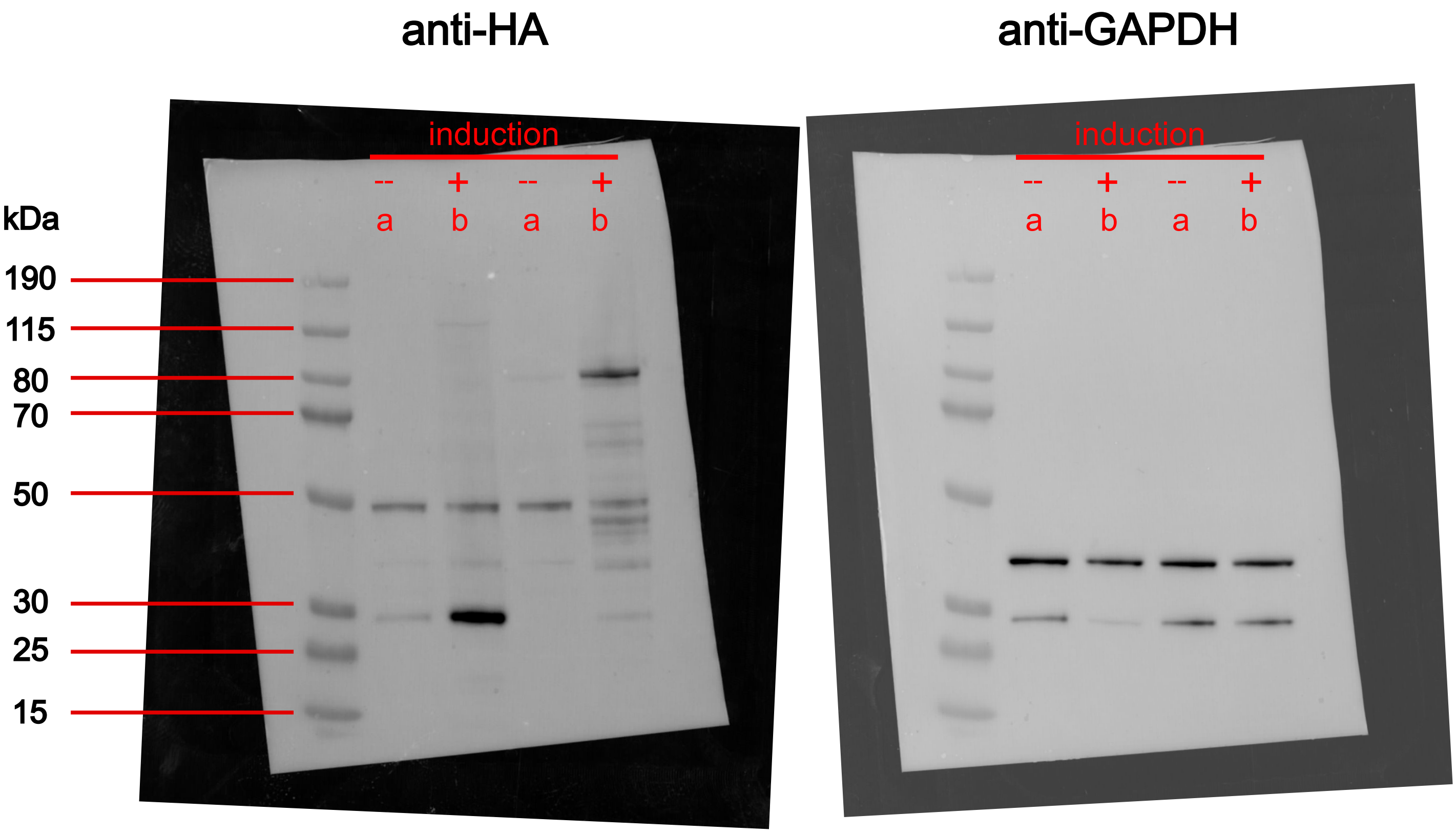

a: *alcA*$_p$*-3×HA-miniTurboID*

b: *alcA*$_p$*-floA-3×HA-miniTurboID*

Raw blot image of Fig. 3b

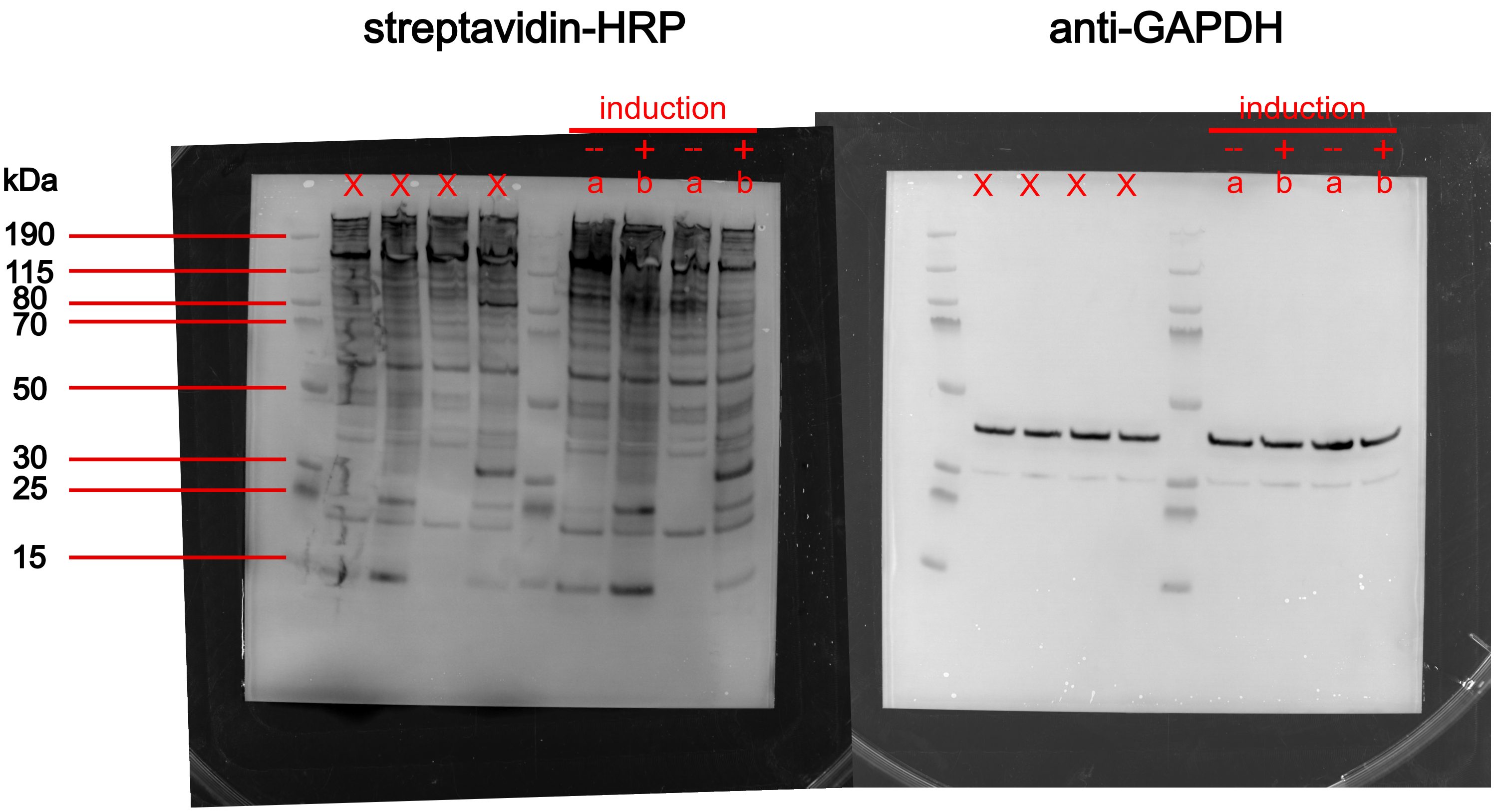

a: *alcA*$_p$*-3×HA-miniTurboID*

b: *alcA*$_p$*-floA-3×HA-miniTurboID*

Raw blot image of Fig. 4a

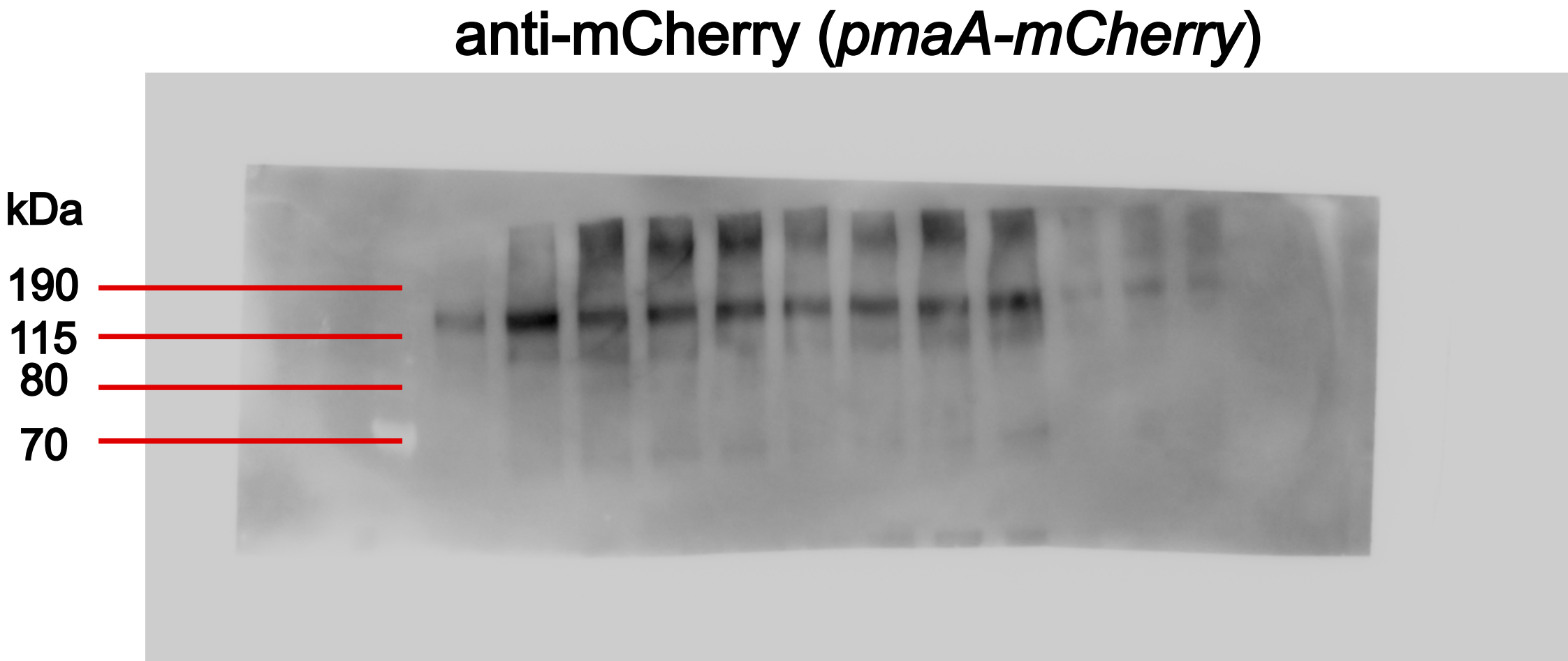

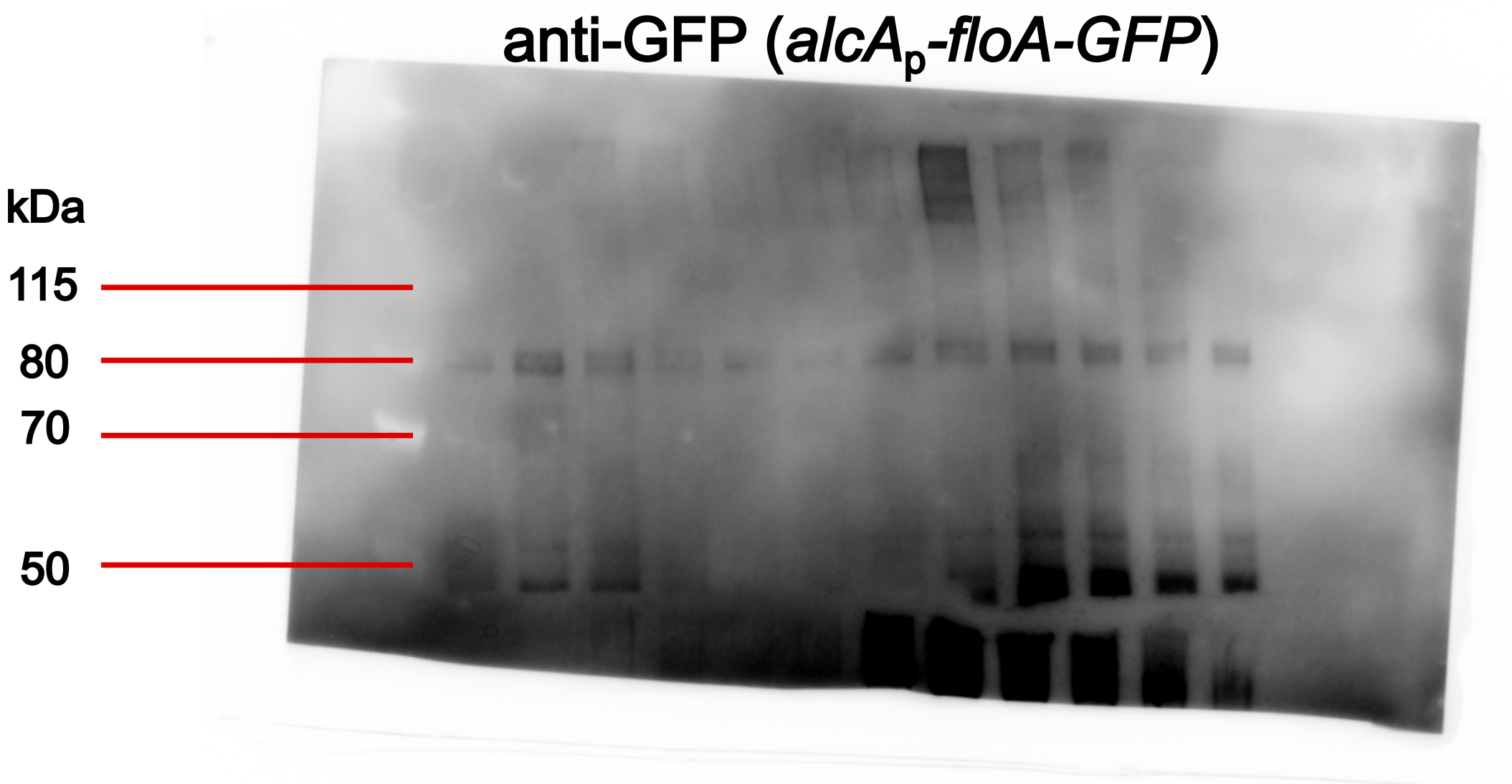

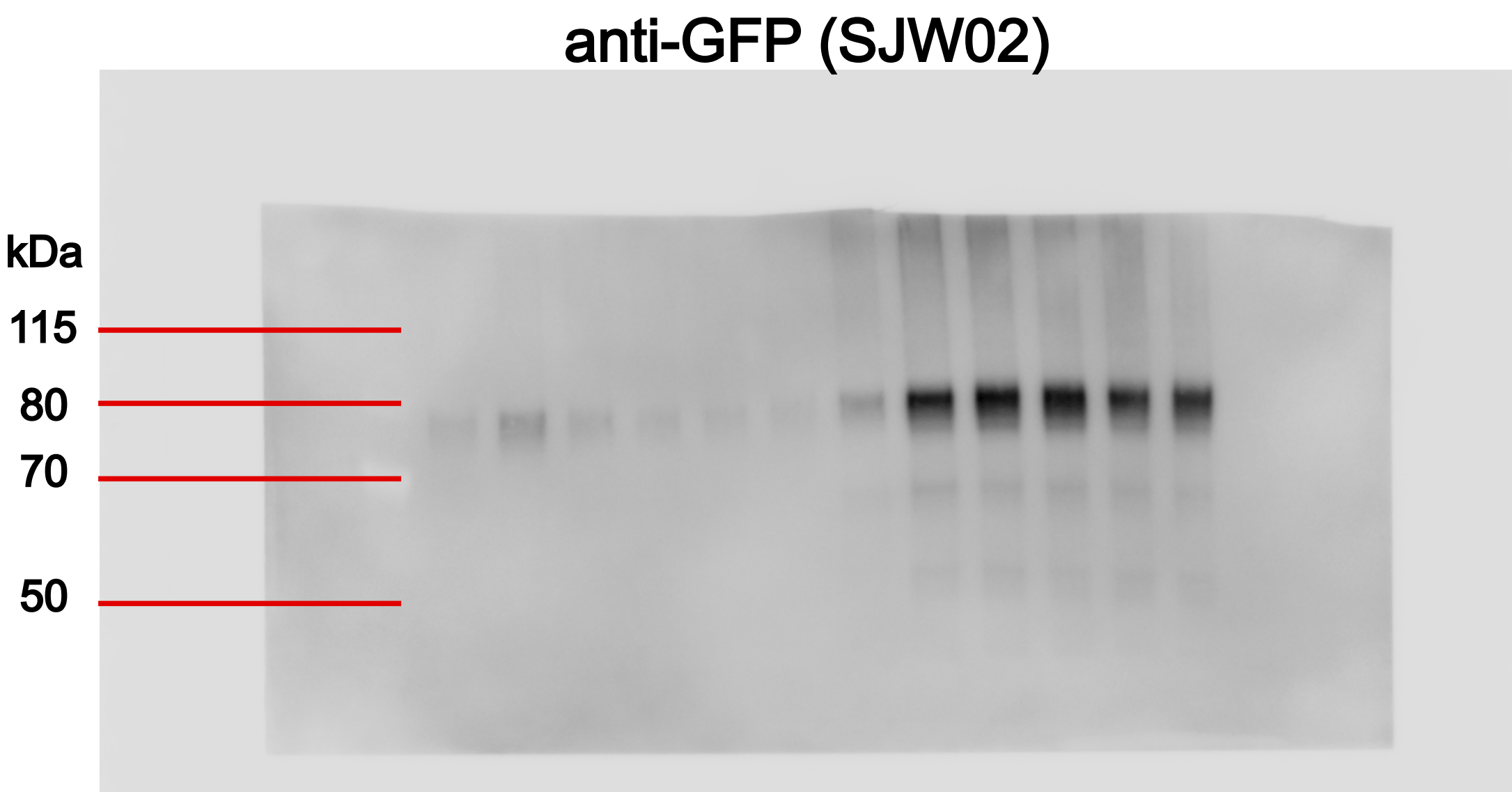

Raw gel image S4 Fig.

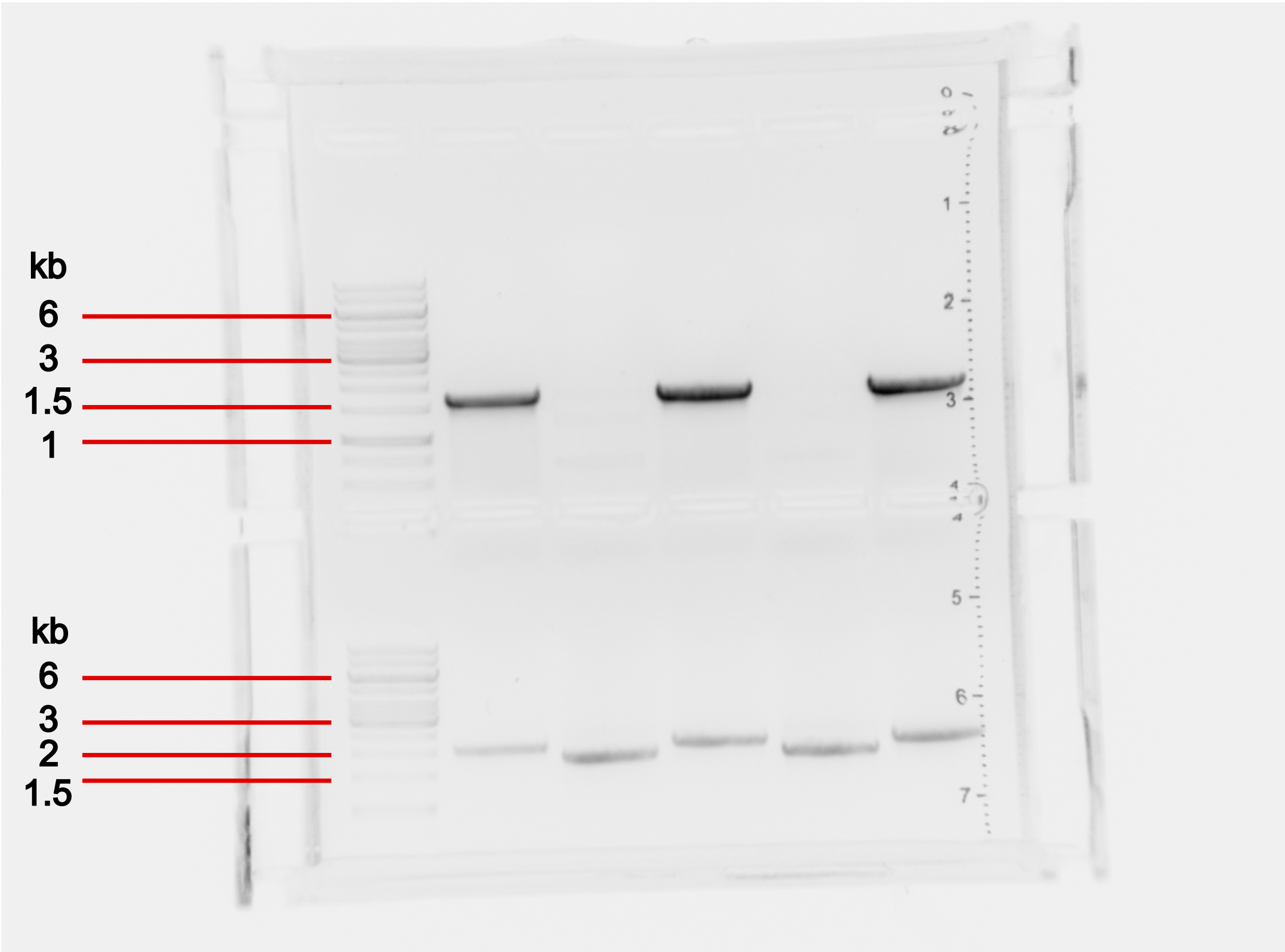

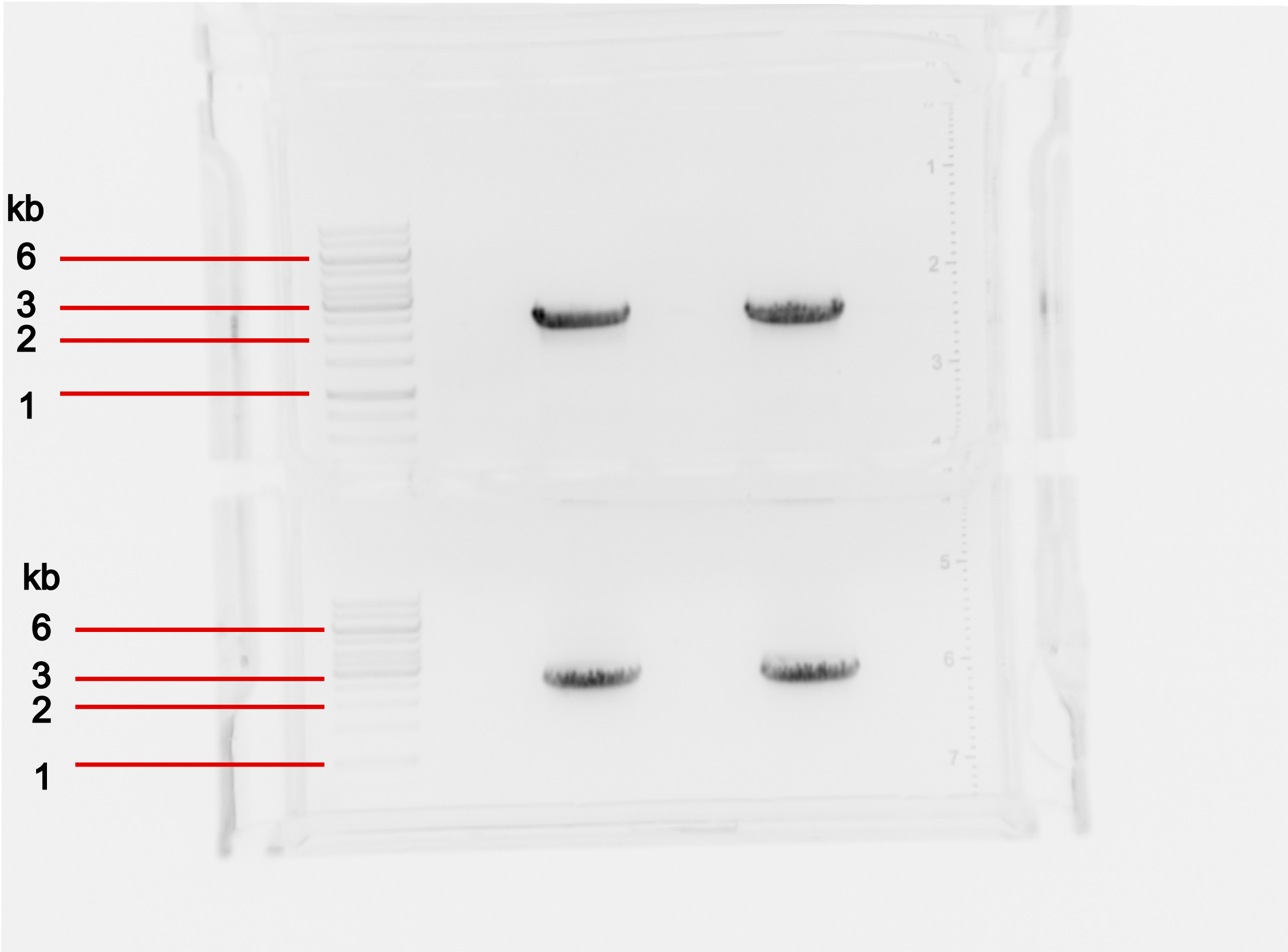

Supplement: Supplementary file 4 [file pgen.1012147.s012.pdf]
