# Supplementary material for: The lipid raft marker flotillin FloA drives relocalization of the plasma membrane H+-ATPase PmaA as a protective response to calcium stress
Source: PLoS Genet. 2026 Oct 29;22(10):e1012147. doi: 10.1371/journal.pgen.1012147 (PMC13734606; doi:10.1371/journal.pgen.1012147)
Supplement: Supplementary file 5 [file pgen.1012147.s014.docx]

## PGENETICS-D-26-00432 Title: The lipid raft marker flotillin FloA of *Aspergillus nidulans* enables relocalization of the plasma membrane H+-ATPase PmaA as part of a protective response to calcium stress

**Authors**: Moemi Kawashima, Thomas Krüger, Maira Rosin, Sophie Tröger-Görler, Thorsten Heinekamp and Axel A. Brakhage

**Response to the reviewers**

We thank the reviewers for their supportive comments and appreciate the suggestions provided. We have addressed each point in detail below. All responses from the authors are marked in blue. References cited in this response follow the numbering used in the revised manuscript.

**Reviewer #1**

This manuscript examines the role of the FloA flotillin protein in A. nidulans to better understand its function in lipid rafts and membrane trafficking. The data indicate that FloA is localized to the plasma membrane, but at sites distinct from the plasma membrane ATPase PmaA. However, a weakness is that it was necessary to overproduce FloA to detect it in these experiments. An interesting aspect of the manuscript is that FLOA expression is induced by calcium and a floA mutant dies more rapidly in the presence of high calcium. Thiscoincides with a failure to internalize the PmaA plasma membrane ATPase. These interesting data were offset by a weakness that the authors did not examine whether PmaA was responsible for the rapid death in the floA mutant. How FloA affects membrane trafficking remains a question since the FloA-GFP was maintained at the plasma membrane under high calcium conditions where PmaA is internalized. Additional experiments would be necessary to make stronger conclusions.

1. A key result in the manuscript is that PmaA-mCherry was not internalized from the plasma membrane in response to high calcium in a floA mutant. The authors conclude that this contributes to the rapid death of the floA mutant in the presence of calcium. However, this is just a correlation. The authors need to present data that supports this conclusion that PmaA is involved. The floA mutant may die more quickly for other reasons.

We thank reviewer #1 for raising this important point regarding the direct involvement of PmaA in the increased cell death of the Δ*floA* mutant under calcium stress. We agree that the original manuscript primarily demonstrated a correlation between impaired PmaA internalization and cell death in the Δ*floA* mutant, rather than establishing a causal relationship.

To strengthen our model, we have added the following results to the revised manuscript:

a. To obtain further evidence that impaired PmaA internalization contributes to the increased cell death of the Δ*floA* mutant, we inhibited PmaA internalization in the wild-type strain using the endocytosis inhibitor latrunculin B during calcium exposure and assessed cell viability (Fig. 6c). Upon treatment with latrunculin B, PmaA-mCherry remained at the plasma membrane during calcium stress, and significantly more cells were positive for Sytox Blue and/or Fluo-4, indicating increased cell death and intracellular calcium accumulation. In contrast, cells exposed to 0.2 M NaCl in the presence of latrunculin B also retained PmaA-mCherry at the plasma membrane but remained largely Sytox Blue negative. These results provide additional evidence that PmaA internalization contributes specifically to the cellular response to calcium stress and supports survival under these conditions.
b. Since the main function of PmaA is to maintain cytosolic pH by extruding protons from the cytosol, we hypothesized that the PmaA internalization leads to cytosolic acidification. Such acidification would alter ion uptake by disruption of the electrochemical gradient as well as dysregulation of voltage-gated channels. To test whether mimicking this physiological consequence of PmaA internalization could improve survival of the ΔfloA mutant, we treated cells with the protonophore CCCP before and during calcium stress (Fig. 7). CCCP treatment significantly reduced the proportion of Sytox Blue-positive cells in the ΔfloA strain. Furthermore, PmaA-mCherry remained at the plasma membrane in both the wild-type and ΔfloA strains following CCCP treatment, indicating that the protective effect of CCCP does not require PmaA internalization. These results further support our model that the physiological consequences associated with reduced PmaA activity at the plasma membrane contribute to protection against calcium stress.

Together, these additional experiments provide evidence for a functional role of PmaA internalization in calcium-stress tolerance and strengthen the mechanistic link between impaired PmaA internalization and the increased sensitivity of the Δ*floA* strain. We thank reviewer #1 again for this comment, which helped us strengthen both the experimental work and the discussion of our proposed model.

2. Does the floA mutation specifically block the internalization of PmaA? Or is the internalization of other proteins blocked too?

We agree with reviewer #1 that this would be an interesting and important question to answer. However, systematically testing whether *floA* deletion affects the internalization of other plasma membrane proteins is challenging, as the conditions that trigger internalization are unknown for many proteins. We therefore focused on the uric acid/xanthine proton symporter UapA, whose internalization upon addition of uric acid has been reported [50]. We hypothesized that, if FloA were required for UapA internalization, deletion of *floA* would increase sensitivity to the toxic purine analog oxypurinol, which is transported by UapA. Inhibition of UapA internalization from the plasma membrane would be expected to maintain oxypurinol uptake and thereby increase toxicity. However, the Δ*floA* strain did not display increased sensitivity to oxypurinol compared with the wild-type strain (S9 Fig.). Although we cannot systematically assess all plasma membrane proteins, this result suggests that FloA is not generally required for the internalization of plasma membrane proteins and may instead facilitate the internalization of specific cargo, such as PmaA.

3. Is FloA-GFP functional? The localization could be affected by the GFP tag, so it is important to show whether FloA-GFP is functional.

We thank reviewer #1 for pointing out the lack of clarity regarding the functionality of the FloA-GFP fusion protein. The localization of FloA-GFP expressed by the control of the *alcA* promoter was previously characterized by Takeshita et al. [30]. They showed that the *alcA*_p_*-floA-GFP* strain exhibits GFP signals in punctate structures at the plasma membrane, with the strongest signals observed in conidia. This localization pattern is consistent with our observations. In addition, Takeshita et al. generated a strain expressing HA-FloA under the control of the endogenous *floA* promoter and demonstrated by indirect immunofluorescence that its localization pattern was comparable to that of the *alcA*_p_*-floA-GFP* strain.

Flotillins undergo post-translational palmitoylation near the N-terminus, which is important for their association with cellular membranes. Since FloA is tagged with GFP at the C-terminus, the tag is unlikely to interfere directly with this N-terminal membrane-targeting mechanism. Together with the comparable localization of HA-FloA reported previously, this supports the conclusion that C-terminal GFP tagging does not substantially alter FloA localization.

Assessing the functionality of the FloA-GFP fusion protein is nevertheless challenging because *floA* is nonessential and its deletion does not result in an obvious growth phenotype under standard conditions. However, the *alcA*_p_-*floA-GFP* strain does not phenocopy the Δ*floA* mutant under calcium stress, supporting the functionality of the fusion protein. To provide additional evidence, we have also included a comparative growth assay of the *alcA*_p_-*floA-GFP* strain and its parental wild-type strain (S5 Fig.). The transgenic strain displayed no obvious growth defect compared with the wild-type strain.

4. The Western blot in Figure 3a is difficult to interpret.

We thank reviewer #1 for pointing out the lack of clarity of Fig. 3a.

a) There seems to be a very limited increase in biotinylated proteins in the lanes where the miniTurboID genes were induced. This raises questions about the efficiency.

When we prepared the figure for this manuscript, we were unfortunately unable to detect strong biotinylation activity of miniTurboID by streptavidin-HRP, despite having successfully detected this signal multiple times previously. We have now resolved this issue by repeating the blot with fresh streptavidin-HRP, and the corresponding figure has been updated accordingly (Fig. 3b).

b) The molecular weight markers are not shown.

We have added molecular weight markers to the figures (Fig. 3a & b).

c) How were the 3xHA-miniTurboID bands detected?

We have detected 3xHA-miniTurboID using an anti-HA antibody. We have added this information in the figure caption.

d) How was GAPDH detected?

We have detected GAPDH using an anti-GAPDH antibody. We have added this information in the figure caption.

e) TurboID-tagged proteins usually autobiotinylate and are the most abundant protein detected on a Western blot probed with Streptavidin. It is not obvious that there are bands detected for miniTurboID proteins.

As explained in (4a), we have resolved this issue using fresh streptavidin-HRP and have now mentioned the enrichment of miniTurboID and FloA-3xHA-miniTurboID in the streptavidin blot in the manuscript.

5. The volcano plot in Figure 3b appears to be skewed toward proteins that were enriched in the FloA-3xHA-miniTurboID over the 3xHA-miniTurboID samples. How were the data sets normalized?

We thank reviewer #1 for this careful observation and for raising the important question of how the proteomics data were normalized. LC-MS/MS analysis after pulldown of biotinylated proteins with streptavidin is generally accompanied by the elution of varying amounts of protein across different sample groups as the amount of biotinylated proteins is inherently different. Consequently, cross-group normalization runs the risk of erasing biological effects. For such samples, cross-group normalization can exceptionally be performed based solely on endogenously biotinylated carboxylases, provided one assumes that they elute consistently across the sample groups—an assumption that does not always necessarily hold true, given that the biotinylated proteins in the samples compete with the artificially biotinylated proteins for binding sites. We have had good results with this approach using human samples, for instance. However, in the case of *A. nidulans*, only pyruvate carboxylase was identified with sufficient abundance, and we did not consider the distribution of variances based on a single protein to be a justifiable basis for normalization. For this reason, we employ group-wise normalization for such samples based on the abundance sum and median. The right skew seems rather inherent to the nature of the sample and is not atypical for BioID or TurboID samples, even with other normalization approaches. Ultimately, we obtain evidence by selecting the proteins with the most pronounced and significant effect sizes and identifying potential interaction partners through background subtraction.

We thank reviewer #1 again for highlighting this point. We hope that this explanation clarifies both our normalization strategy and the origin of the observed distribution in the volcano plot.

6. The proteins presented in Table 1 are all very abundant proteins. This raises concern that they are being detected because they are abundant, not necessarily because they are in proximity to FloA. Furthermore, most of the proteins are not plasma membrane proteins – is it likely they would interact with FloA?

We thank reviewer #1 for highlighting this important point. Although flotillin is a membrane protein, it is thought to localize on the inner leaflet of the lipid bilayer, where it can interact with cytosolic proteins as well as membrane proteins. Therefore, it is not unexpected that many of the BioID hits are cytosolic proteins. Moreover, many of the BioID hits are involved in the endocytic pathway, suggesting that the proteins have some functional relevance to each other. To clarify the relationship between the proteins detected by BioID, we have added a figure which depicts their subcellular localizations (Fig. 3d).

We also agree that the high abundance of some proteins could increase their likelihood of being detected. However, candidate proteins were not selected based on absolute abundance alone, but rather on their enrichment in the FloA-3xHA-miniTurboID samples relative to the 3xHA-miniTurboID control and their statistical significance. Thus, the analysis specifically aimed to distinguish proteins enriched in proximity to FloA from proteins detected as part of the general background.

7. PmaA is listed in Table 1 as being in proximity to FloA, but this is not supported by other data. The proteins appear to reside in different compartments in the plasma membrane and FloA stays at the plasma membrane when PmaA is internalized in the presence of high calcium.

We agree with reviewer #1 that the detection of PmaA as a hit in the BioID experiment, despite the lack of apparent colocalization between PmaA-mCherry and FloA-GFP, raises an important question. However, one advantage of BioID over other methods to investigate protein-protein interactions, such as co-immunoprecipitation, is its ability to capture transient interactions. We therefore propose that FloA may interact only transiently with PmaA, without forming a stable complex detectable by conventional colocalization analyses. This interpretation is also consistent with a model in which flotillins facilitate endocytosis, rather than functioning as core components of the endocytic machinery or as cargo proteins themselves.

To further clarify this point, we have expanded the Discussion to include recent structural findings on mammalian flotillins. Human flotillin-1 and flotillin-2 were recently shown to assemble into a membrane-associated 44-mer with a dome-shaped architecture that defines a confined membrane domain and may contribute to the sequestration and organization of membrane proteins [82]. Although it remains unknown whether fungal flotillins form such structures, these findings provide a plausible structural basis for our proposed “clustering assistance” model, in which FloA remains associated with the plasma membrane while transiently organizing PmaA into membrane domains that facilitate its subsequent internalization.

8. The FloA-3xHA-miniTurboID BioID data presented in Table 1 should include standard deviations for the fold enrichment in the different assays.

We have revised Table 1 to include the mean LFQ intensities together with their corresponding standard deviations for the BioID samples. We believe this addition provides a clearer representation of the variability across the replicate measurements.

Minor comment

Line 108 change to Figure 2b?

Thank you for pointing out this error. We have corrected the reference to Fig. 2b.

**Reviewer #2**

The main subject of this manuscript is the “Role of theFlotillin Protein FloA in the Relocalization of the H+-ATPase PmaA in the Cell Membrane as a Protective Mechanism Against Calcium Stress”
The authors use the filamentous fungus Aspergillus nidulans as an experimental system to investigates the flotillin protein FloA in the organization of membranes during calcium stress. The floA gene is barely expressed during regular growth, but its expression specifically increases thousandfold during calcium stress, but not during other osmotic stressors. Using fluorescence and light microscopy, detergent resistant membrane (DRM) isolation, Western blot analysis, and proximity-dependent biotin identification (BioID) analysis, the authors present several remarkable results.

For example, the manuscript demonstrates that the FloA protein interacts with diverse endocytosis proteins. Further, the authors demonstrate that FloA is enriched together with the plasma membrane H+-ATPase PmaA in lipid rafts. During calcium stress,PmaA relocates FloA dependent from the membrane system into intracellular vesicles.

The strength of this manuscript is the high technical standards and the conclusion from their data resulting in a well-developed model explaining how stress signals trigger upregulation of floA expression, and how FloA protein binds clathrin, actin and myosin V.
Finally, the authors propose a model wherein the controlled degradation sequestration of PmaA protects the hyphae from calcium-induced cell death. FloA likely act at the cytoplasmic membrane by mediating pre-endocytic clustering of PmaA rather than participating directly in the endocytic machinery (Fig. 7). Notably, FloA is required for PmaA internalization under calcium stress, thereby promoting cell survival. Overall, this manuscript proves A. nidulans to be a powerful model system for dissecting the functions of members of the stomatin/prohibitin/flotillin/HflK/C (SPFH) domain-containing protein family from eukaryoticcells, paving the way for novel antifungal strategies that target the essential plasma membrane H+-ATPase. In my opinion, this very carefully prepared manuscript is well within the scope of the Journal.

Minor point:

“floA epression” should consistently be used with a lowercase letter (e.g. line 111,185, 233), while the FloA protein has be used with a capital letter.

We thank reviewer #2 for their time and effort in reviewing this manuscript. We have edited *floA* to have lowercase when denoting the gene and uppercase when denoting the protein.

**Reviewer #3**

In this manuscript Kawashima et al., characterized the role of a lipid raft flotillin protein, FloA, in Aspergillus nidulans. Key findings of the current study include:

1. FloA expression is upregulated in the presence of calcium stress.
2. FloA is located in punctate structures on the membrane and interacted with proteins involved in endocytosis.

3. FloA majorly interacted with the plasma membrane ATPase PmaA and regulated its internalization from the cytoplasmic membrane under calcium stress. Through the deletion ofFloA the authors show FloA-dependent PmaA internalization to control calcium stress induced toxicity. In the absence of FloA PmaA internalization is abolished indicating the role of a lipid raft protein in regulating intracellular calcium overload.
Overall, this is an interesting mechanistic study deciphering the role of a lipid raft protein in the regulation of calcium via a plasma membrane protein. I have few specific comments that the authors should be able to address.

Specific Comments:

1. Calcium stress response links to flotillin FloA is very interesting. This is particularly important under antifungal treatments, such as in the presence of azoles. Does PmaA internalize under other conditions that induce calcium stress?

We thank reviewer #3 for this important question. One limitation of this study is that the simple addition of calcium to the growth medium cannot induce the expression of *floA.* . We initially hypothesized that this was caused by the presence of magnesium in the medium, as magnesium inhibited calcium-dependent *floA* induction (S8 Fig). However, removal of magnesium did not restore *floA* induction upon calcium exposure, indicating that this effect cannot be attributed to magnesium alone. Our results therefore suggest that additional ionic components of the medium interfere with calcium-dependent regulation of *floA*. We have included these data in the Supporting Information (S8 Fig).

Probably due to such interference, we do not see a growth defect of the *floA* deletion mutant grown on AMM+0.2 M CaCl_2_ agar plates. However, we have added photos of the wild-type strain and the *floA* deletion mutant grown on AMM agar plates with caspofungin, voriconazole, amphotericin B and CaCl_2_ as well as heat stress at 42 °C for further characterization of the deletion mutant (S7 Fig.).

2. As flotillin is a lipid raft protein I was wondering if the floA deletion strain showed differences in lipid raft distribution. Probably filipin staining can be done to visualize changes.

We thank reviewer #3 for this insightful suggestion. Deletion of *floA* has previously been reported to affect the integrity of sterol-rich membrane domains at hyphal tips, as filipin staining was more frequently dispersed into subapical regions in the Δ*floA* strain [30]. We therefore assessed the effect of *floA* deletion on ergosterol distribution under both control conditions (AMM) and calcium stress (0.2 M CaCl_2_) (S11 Fig.).

Under control conditions, filipin signals were relatively homogeneously distributed along the plasma membrane in both the wild-type and Δ*floA* strains. In contrast, under calcium stress, filipin staining appeared more dispersed in the Δ*floA* strain than in the wild-type strain. This suggests that the effect of *floA* deletion on sterol organization becomes more pronounced under conditions in which *floA* is strongly induced. We thank reviewer #3 for suggesting this experiment, which provided additional insight into the role of FloA in membrane organization under calcium stress.

3. Lines 106-108: I think the authors are referring to Fig.2b here. 0.2 M CaCl2 shows increased fluorescence, but the figure is not that clear. Some quantitative analysis here would be helpful.

We agree with reviewer #3 that the increased intensity of the GFP signals in Fig. 2b is unclear. We have updated the figure to a microscopic image which clearly indicates the upregulation of *floA-GFP* under calcium stress. We have also corrected the reference to Fig 2b.

4. Mammalian membranes are significantly different from fungal membranes in their composition. Have the authors looked to see if FloA deletion mutant can be complemented with any one of the mammalian flotillin paralogs. This would be quite interesting to know.

We thank reviewer #3 for this thoughtful suggestion. We agree that complementation with a mammalian paralog would be an interesting experiment to assess the functional conservation of flotillins across distant eukaryotic lineages. However, a cross-kingdom complementation experiment would require substantial additional optimization and validation that we feel is beyond the scope of the present work. We have revised the Discussion to acknowledge this possibility and to highlight it as an interesting direction for future work.

5. Lines 120-122: While this is very interesting difference noted between A. nidulans and A. fumigatus flotillin, I recommend including more details on the homology of FloA between A. nidulans and A. fumigatus.

We thank reviewer #3 for this suggestion. We have added details on the sequence similarity of FloA between *A. nidulans* and *A. fumigatus*. Furthermore, we performed an analysis of the 1 kb promoter region of *floA* in both organisms and found three putative CrzA-binding motifs in *A. fumigatus*, whereas none were identified in *A. nidulans*. We believe that these additional analyses have strengthened the discussion by providing further insight into the conserved and species-specific aspects of FloA regulation.
