# Supplementary material for: The lipid raft marker flotillin FloA drives relocalization of the plasma membrane H+-ATPase PmaA as a protective response to calcium stress
Source: PLoS Genet. 2026 Oct 29;22(10):e1012147. doi: 10.1371/journal.pgen.1012147 (PMC13734606; doi:10.1371/journal.pgen.1012147)
Supplement: Supplementary file 6 [file pgen.1012147.s015.docx]

**Response to the reviewers**

We sincerely thank the editors for their careful evaluation of the manuscript. We are very encouraged by the overall positive assessment and the recognition of the improvements made in the previous revision. We are also grateful for the further suggestions made by reviewer #1 and have commented on them below.

All responses from the authors are marked in blue.

Reviewer #1: The authors made significant changes to the revised manuscript that have improved the support for their conclusions. However, there are still some concerns that need to be addressed.


1. Page 9, Figure 3. GO term analysis should be presented to show whether there was a statistically significant enrichment of endocytic proteins.

We thank reviewer #1 for this comment. To address this point, we performed both GO and KEGG enrichment analyses using the complete set of proteins identified in the BioID experiment. Neither analysis revealed significant enrichment of endocytosis- or endosome-related terms/pathway.

We have revised the manuscript to ensure that the text only states the identification of several FloA-proximal proteins with known roles in endocytosis and not the enrichment of the endocytic pathway. We have indicated the changes below and now describe this observation more conservatively.

Line 159: We have changed the term “physical interaction” with “proximity”. We feel this is more appropriate as BioID is only a screening method and the direct physical interaction must be validated using other methods such as yeast-two-hybrid or co-immunoprecipitation.

Line 186: We have replaced “plays a role in” with “may have an influence on”.

2. Table 1. Define what is meant by LQF intensities. What are the units for the values reported as the “mean”. Is it reported as log2? Or as raw counts?

LFQ stands for "label-free quantification". LFQ intensities are by definition a dimensionless quantity that can best be expressed as detector counts per second (cps), *i.e*., the peak intensity of peptide ion signals (on the peptide level) and the sum of the peak intensities of all quantified peptides of the respective proteins (on the protein level). The values are always log2-transformed. Logarithmic transformation stabilizes the variance across the entire measurement range, as raw protein intensities in mass spectrometry often span several orders of magnitude and the variance typically increases in proportion to the mean (heteroscedastic data). The mean value is the mean of the protein abundances of all replicates of the same sample group.

We have added explanations to the acronyms “LFQ” and “SD” in the Table 1 legend for further clarification.

3. Figure 4A. The label “raft” under the blots should be changed to DRM, since that is what was analyzed. As the authors pointed out, they are not always the same.

We agree with reviewer #1 that the labels in the figure may be misleading and have therefore changed the “raft” and “non-raft” labels to “DRM” and “DSM (detergent soluble membrane)”, respectively. We have also added explanations to these terms in the main text (line 216).

4, Line 173 states that 111 proteins were significantly enriched compared to the negative control. The authors should state what is the negative control in this case. Also, it was stated on line 172 that 270 proteins were uniquely detected in the floA-3xHA-miniTurboID strain. This is confusing.
We agree with reviewer #1 that the two statements appear contradictory. This discrepancy is due to the different criteria used for the two analyses. For the enrichment analysis relative to the control (3xHA-miniTurboID), we only included proteins detected in at least five of the six biological replicates. In contrast, no such threshold was applied when identifying uniquely detected proteins.

To avoid confusion, we have removed the statement regarding the number of uniquely detected proteins from the manuscript. We have also changed “negative control” in line 173 to “3xHA-miniTurboID strain”.

5. Line 222. Pma1-cherry and FloA-GFP do not look punctate. They seem relatively evenly distributed in the membrane. The authors should present an airyscan image of the top of the cell to provide support for their conclusion that these proteins are in puncta.

We agree with reviewer #1 that the term “punctate” lacks accuracy as both but especially PmaA-mCherry have inhomogeneities in the distribution along the plasma membrane but do not necessarily have dotted structures. We have changed the main text accordingly (line 231). We believe the new description reflects the observation more accurately and thank reviewer #1 for raising this point.

6. Line 276. The authors state that “a substantial fraction of conidia failed to internalize PmaA-mCherry”. This should be quantified and reported as the percent of cells that failed to internalize PmaA-mCherry and it should also be stated how many independent experiments were carried out.

We thank reviewer #1 for this important comment. We agree that quantification of this observation would strengthen our report and have added this data as Fig. 6b. Details on the statistics can be found in the figure legend.

7. Figure S9. The sensitivity to oxypurinol provides some indirect evidence. However, it would have been more compelling to see if UapA-GFP or any other plasma membrane protein stayed on the membrane in the floA mutant treated with Calcium, or if it was internalized like Pma1.

We thank reviewer #1 for this discussion point and may consider this suggestion in a follow-up study.

8. Define the floA-C strain shown in Figure 6. I assume it is the complemented strain? This should be described in the Figure Legend.

We thank reviewer #1 for pointing out the lack of clarity. We have stated in the figure legend that the *floA^c^* strain in Fig. 6 indicates the *floA* complementation strain.

9. Line 342 Define “SPFH family”
We thank reviewer #1 for this hint. “SPFH” had been defined in line 64 upon first appearance.

Minor comments:
-The term knockout always seems like lab jargon to me. It would be clearer to say deletion mutant or insertion mutant, as this better describes the type of “knockout” mutation. However, I leave it to the authors if they want to make this change or not.

Thank you for this important discussion point. This is a matter of debate. We feel that we should stick to the term knockout which we have previously used in several other publications.

-Line 130-131. The text states that there are three CrzA-binding motifs in Af floA, but Fig S6 shown only two motifs.

We thank reviewer #1 for raising this point. We have adjusted the figure so that the two arrows which were overlapping can be distinguished better.

Reviewer #2: Dear authors, Thank you for providing your revised and improved ms. With that, i can recommend publication of the ms

Thank you very much for your helpful and positive assessment of our study.

Reviewer #3: This version of the manuscript is improved by the addition of new data on FloA. I do not have any further comments on the manuscript.

Thank you very much for your helpful and positive assessment of our study.
