# Supplementary material for: The lipid raft marker flotillin FloA drives relocalization of the plasma membrane H+-ATPase PmaA as a protective response to calcium stress
Source: PLoS Genet. 2026 Oct 29;22(10):e1012147. doi: 10.1371/journal.pgen.1012147 (PMC13734606; doi:10.1371/journal.pgen.1012147)
Supplement: Peer review history

# Peer review: Decision Letter 0

**DOI:** 10.1371/journal.pgen.1012147.r001

**Authors:** Gustavo Goldman, Benjamin Schwessinger

10 Jun 2026

PGENETICS-D-26-00432

The lipid raft marker flotillin FloA drives relocalization of the plasma membrane H^+^-ATPase PmaA as a protective response to calcium stress

PLOS Genetics

Dear Dr. Brakhage,

Thank you for submitting your manuscript to PLOS Genetics. After careful consideration, we feel that it has merit but does not fully meet PLOS Genetics's publication criteria as it currently stands. Therefore, we invite you to submit a revised version of the manuscript that addresses the points raised during the review process.

Please submit your revised manuscript by Aug 09 2026 11:59PM. If you will need more time than this to complete your revisions, please reply to this message or contact the journal office at plosgenetics@plos.org. When you're ready to submit your revision, log on to https://www.editorialmanager.com/pgenetics/ and select the 'Submissions Needing Revision' folder to locate your manuscript file.

Please include the following items when submitting your revised manuscript:

* A letter that responds to each point raised by the editor and reviewer(s). You should upload this letter as a separate file labeled 'Response to Reviewers'. This file does not need to include responses to any formatting updates and technical items listed in the 'Journal Requirements' section below.

* A marked-up copy of your manuscript that highlights changes made to the original version. You should upload this as a separate file labeled 'Revised Manuscript with Track Changes'.

* An unmarked version of your revised paper without tracked changes. You should upload this as a separate file labeled 'Manuscript'.

If you would like to make changes to your financial disclosure, competing interests statement, or data availability statement, please make these updates within the submission form at the time of resubmission. Guidelines for resubmitting your figure files are available below the reviewer comments at the end of this letter.

As the corresponding author, your ORCID iD is verified in the submission system and will appear in the published article. PLOS supports the use of ORCID, and we encourage all coauthors to register for an ORCID iD and use it as well. Please encourage your coauthors to verify their ORCID iD within the submission system before final acceptance, as unverified ORCID iDs will not appear in the published article. Only the individual author can complete the verification step; PLOS staff cannot verify ORCID iDs on behalf of authors.

We look forward to receiving your revised manuscript.

Kind regards,

Gustavo Henrique Goldman, Ph.D.

Guest Editor

PLOS Genetics

Benjamin Schwessinger

Section Editor

PLOS Genetics

Aimée Dudley

Editor-in-Chief

PLOS Genetics

Anne Goriely

Editor-in-Chief

PLOS Genetics

**Additional Editor Comments:**

The manuscript has now been reviewed by three experts in the field. As you will see, the reviewers find the central question—how the flotillin protein FloA regulates PmaA internalization under calcium stress—potentially interesting and timely. However, all three reviewers raise important concerns that currently preclude acceptance.

Reviewer 1 identifies several substantive, technical, and conceptual weaknesses. Chief among these is the correlative conclusion that failed PmaA internalization causes the rapid death of the floA mutant under high calcium; direct evidence linking PmaA to this lethality is required. Additionally, this reviewer asks whether the internalization block is specific to PmaA or affects other cargo, whether FloA-GFP is functional, and points to concerns regarding the BioID data (efficiency, normalization, interpretation of abundant versus proximate proteins, and lack of standard deviations). The apparent discrepancy between BioID suggesting proximity of FloA and PmaA and microscopy showing distinct membrane compartments also needs to be addressed.

Reviewer 2 is broadly positive, highlighting the technical quality and the well-developed model. Only minor editorial points are raised.

Reviewer 3 finds the study mechanistically interesting but requests additional experiments and clarifications, including testing whether PmaA internalization occurs under other calcium stress conditions, examining lipid raft distribution by filipin staining in the floA mutant, quantifying the calcium-induced fluorescence shown in Figure 2b, exploring cross-species complementation with mammalian flotillins, and providing more homology details between A. nidulans and A. fumigatus.

Given the requirement for new experiments to address the concerns of Reviewers 1 and 3, particularly the need to establish causality between PmaA retention and calcium sensitivity, I cannot accept the manuscript in its current form.

I am therefore offering you the opportunity to revise your manuscript to address all of the above points. If you can provide compelling additional data—especially to test whether PmaA directly mediates the rapid death phenotype (Reviewer 1, point 1) and to address the specificity of the internalization block (Reviewer 1, point 2)—the manuscript could become suitable for publication in PLOS Genetics.

Please submit your revised version by [date], along with a point-by-point response to each reviewer comment.

**Journal Requirements:**

1)  Please ensure that the CRediT author contributions listed for every co-author are completed accurately and in full.

At this stage, the following Authors/Authors require contributions: Moemi Kawashima, Thomas Krüger, Maira Rosin, Sophie Tröger-Görler, Thorsten Heinekamp, and Axel A. Brakhage. Please ensure that the full contributions of each author are acknowledged in the "Add/Edit/Remove Authors" section of our submission form.

The list of CRediT author contributions may be found here: https://journals.plos.org/plosgenetics/s/authorship#loc-author-contributions

2) We ask that a manuscript source file is provided at Revision. Please upload your manuscript file as a .doc, .docx, .rtf or .tex. If you are providing a .tex file, please upload it under the item type u2018LaTeX Source Fileu2019 and leave your .pdf version as the item type u2018Manuscriptu2019.

3) Please provide an Author Summary. This should appear in your manuscript between the Abstract (if applicable) and the Introduction, and should be 150-200 words long. The aim should be to make your findings accessible to a wide audience that includes both scientists and non-scientists. Sample summaries can be found on our website under Submission Guidelines:

https://journals.plos.org/plosgenetics/s/submission-guidelines#loc-parts-of-a-submission

NOTE: Currently you have labeled it as " Author Abstract"

4) Please upload all main figures as separate Figure files in .tif or .eps format. For more information about how to convert and format your figure files please see our guidelines:

https://journals.plos.org/plosgenetics/s/figures

5) Please upload a copy of Figure 1 to 7 which you refer to in your text on the manuscript file. Or, if the figure is no longer to be included as part of the submission please remove all reference to it within the text.

6) Thank you for stating "The mass spectrometry proteomics data have been deposited to the ProteomeXchange Consortium via the PRIDE partner repository with the dataset identifier PXD076500 and 10.6019/PXD076500."

We strongly recommend all authors deposit their data before acceptance, as the process can be lengthy and hold up publication timelines. Please note that, though access restrictions are acceptable now, your entire minimal dataset will need to be made freely accessible if your manuscript is accepted for publication. This policy applies to all data except where public deposition would breach compliance with the protocol approved by your research ethics board. If you are unable to adhere to our open data policy, please kindly revise your statement to explain your reasoning and we will seek the editor's input on an exemption.

7) Please ensure that the funders and grant numbers match between the Financial Disclosure field and the Funding Information tab in your submission form. Note that the funders must be provided in the same order in both places as well. Currently, " M.K. and S.T.G. were also supported by the DFG Excellence Graduate School “Jena School for Microbial

Communication” is missing from the Funding Information

8) Please amend your detailed Financial Disclosure statement. This is published with the article, therefore should be completed in full sentences and contain the exact wording you wish to be published.

i). State what role the funders took in the study. If the funders had no role in your study, please state: “The funders had no role in study design, data collection and analysis, decision to publish, or preparation of the manuscript.”

ii). If any authors received a salary from any of your funders, please state which authors and which funders.

**Reviewers' comments:**

Reviewer's Responses to Questions

**Comments to the Authors:**

**Please note here if the review is uploaded as an attachment.**

Reviewer #1: This manuscript examines the role of the FloA flotillin protein in A. nidulans to better understand its function in lipid rafts and membrane trafficking. The data indicate that FloA is localized to the plasma membrane, but at sites distinct from the plasma membrane ATPase PmaA. However, a weakness is that it was necessary to overproduce FloA to detect it in these experiments. An interesting aspect of the manuscript is that FLOA expression is induced by calcium and a floA mutant dies more rapidly in the presence of high calcium. This coincides with a failure to internalize the PmaA plasma membrane ATPase. These interesting data were offset by a weakness that the authors did not examine whether PmaA was responsible for the rapid death in the floA mutant. How FloA affects membrane trafficking remains a question since the FloA-GFP was maintained at the plasma membrane under high calcium conditions where PmaA is internalized. Additional experiments would be necessary to make stronger conclusions.

1. A key result in the manuscript is that PmaA-mCherry was not internalized from the plasma membrane in response to high calcium in a floA mutant. The authors conclude that this contributes to the rapid death of the floA mutant in the presence of calcium. However, this is just a correlation. The authors need to present data that supports this conclusion that PmaA is involved. The floA mutant may die more quickly for other reasons.

2. Does the floA mutation specifically block the internalization of PmaA? Or is the internalization of other proteins blocked too?

3. Is FloA-GFP functional? The localization could be affected by the GFP tag, so it is important to show whether FloA-GFP is functional.

4. The Western blot in Figure 3a is difficult to interpret.

a) There seems to be a very limited increase in biotinylated proteins in the lanes where the miniTurboID genes were induced. This raises questions about the efficiency.

b) The molecular weight markers are not shown.

c) How were the 3xHA-miniTurboIDm bands detected?

d) How was GAPDH detected?

e) TurboID-tagged proteins usually autobiotinylate and are the most abundant protein detected on a Western blot probed with Streptavidin. It is not obvious that there are bands detected for miniTurboID proteins.

5. The volcano plot in Figure 3b appears to be skewed toward proteins that were enriched in the FloA-3xHA-miniTurboID over the 3xHA-miniTurboID samples. How were the data sets normalized?

6. The proteins presented in Table 1 are all very abundant proteins. This raises concern that they are being detected because they are abundant, not necessarily because they are in proximity to FloA. Furthermore, most of the proteins are not plasma membrane proteins – is it likely they would interact with FloA?

7. PmaA is listed in Table 1 as being in proximity to FloA, but this is not supported by other data. The proteins appear to reside in different compartments in the plasma membrane and FloA stays at the plasma membrane when PmaA is internalized in the presence of high calcium.

8. The FloA-3xHA-miniTurboID BioID data presented in Table 1 should include standard deviations for the fold enrichment in the different assays.

Minor comment

Line 108 change to Figure 2b?

Reviewer #2: KawashimaEtal PlosGenetics2026

The main subject of this manuscript is the “Role of the Flotillin Protein FloA in the Relocalization of the H+-ATPase PmaA in the Cell Membrane as a Protective Mechanism Against Calcium Stress”

The authors use the filamentous fungus Aspergillus nidulans as an experimental system to investigates the flotillin protein FloA in the organization of membranes during calcium stress. The floA gene is barely expressed during regular growth, but its expression specifically increases thousandfold during calcium stress, but not during other osmotic stressors. Using fluorescence and light microscopy, detergent resistant membrane (DRM) isolation, Western blot analysis, and proximity-dependent biotin identification (BioID) analysis, the authors present several remarkable results.

For example, the manuscript demonstrates that the FloA protein interacts with diverse endocytosis proteins. Further, the authors demonstrate that FloA is enriched together with the plasma membrane H+-ATPase PmaA in lipid rafts. During calcium stress, PmaA relocates FloA dependent from the membrane system into intracellular vesicles.

The strength of this manuscript is the high technical standards and the conclusion from their data resulting in a well-developed model explaining how stress signals trigger upregulation of floA expression, and how FloA protein binds clathrin, actin and myosin V.

Finally, the authors propose a model wherein the controlled degradation sequestration of PmaA protects the hyphae from calcium-induced cell death. FloA likely act at the cytoplasmic membrane by mediating pre-endocytic clustering of PmaA rather than participating directly in the endocytic machinery (Fig. 7). Notably, FloA is required for PmaA internalization under calcium stress, thereby promoting cell survival. Overall, this manuscript proves A. nidulans to be a powerful model system for dissecting the functions of members of the stomatin/prohibitin/flotillin/HflK/C (SPFH) domain-containing protein family from eukaryotic cells, paving the way for novel antifungal strategies that target the essential plasma membrane H+-ATPase. In my opinion, this very carefully prepared manuscript is well within the scope of the Journal.

Minor point:

“floA epression” should consistently be used with a lowercase letter (e.g. line 111,185, 233), while the FloA protein has be used with a capital letter.

Reviewer #3: General Comments:

In this manuscript Kawashima et al., characterized the role of a lipid raft flotillin protein, FloA, in Aspergillus nidulans. Key findings of the current study include:

1. FloA expression is upregulated in the presence of calcium stress.

2. FloA is located in punctate structures on the membrane and interacted with proteins involved in endocytosis.

3. FloA majorly interacted with the plasma membrane ATPase PmaA and regulated its internalization from the cytoplasmic membrane under calcium stress. Through the deletion of FloA the authors show FloA-dependent PmaA internalization to control calcium stress induced toxicity. In the absence of FloA PmaA internalization is abolished indicating the role of a lipid raft protein in regulating intracellular calcium overload.

Overall, this is an interesting mechanistic study deciphering the role of a lipid raft protein in the regulation of calcium via a plasma membrane protein. I have few specific comments that the authors should be able to address.

Specific Comments:

1. Calcium stress response links to flotillin FloA is very interesting. This is particularly important under antifungal treatments, such as in the presence of azoles. Does PmaA internalize under other conditions that induce calcium stress?

2. As flotillin is a lipid raft protein I was wondering if the floA deletion strain showed differences in lipid raft distribution. Probably filipin staining can be done to visualize changes.

3. Lines 106-108: I think the authors are referring to Fig.2b here. 0.2 M CaCl2 shows increased fluorescence, but the figure is not that clear. Some quantitative analysis here would be helpful.

4. Mammalian membranes are significantly different from fungal membranes in their composition. Have the authors looked to see if FloA deletion mutant can be complemented with any one of the mammalian flotillin paralogs. This would be quite interesting to know.

5. Lines 120-122: While this is very interesting difference noted between A. nidulans and A. fumigatus flotillin, I recommend including more details on the homology of FloA between A. nidulans and A. fumigatus.

**********

**Have all data underlying the figures and results presented in the manuscript been provided?**

Large-scale datasets should be made available via a public repository as described in the *PLOS Genetics*  data availability policy, and numerical data that underlies graphs or summary statistics should be provided in spreadsheet form as supporting information.

Reviewer #1: Yes

Reviewer #2: Yes

Reviewer #3: Yes

**********

PLOS authors have the option to publish the peer review history of their article (what does this mean?). If published, this will include your full peer review and any attached files.

If you choose “no”, your identity will remain anonymous but your review may still be made public.

**Do you want your identity to be public for this peer review?**  For information about this choice, including consent withdrawal, please see our Privacy Policy.

Reviewer #1: No

Reviewer #2: No

Reviewer #3: No

[NOTE: If reviewer comments were submitted as an attachment file, they will be attached to this email and accessible via the submission site. Please log into your account, locate the manuscript record, and check for the action link "View Attachments". If this link does not appear, there are no attachment files.]

**Figure resubmission:**

While revising your submission, we strongly recommend that you use PLOS’s NAAS tool (https://ngplosjournals.pagemajik.ai/artanalysis) to test your figure files. NAAS can convert your figure files to the TIFF file type and meet basic requirements (such as print size, resolution), or provide you with a report on issues that do not meet our requirements and that NAAS cannot fix.

After uploading your figures to PLOS’s NAAS tool - https://ngplosjournals.pagemajik.ai/artanalysis, NAAS will process the files provided and display the results in the "Uploaded Files" section of the page as the processing is complete. If the uploaded figures meet our requirements (or NAAS is able to fix the files to meet our requirements), the figure will be marked as "fixed" above. If NAAS is unable to fix the files, a red "failed" label will appear above. When NAAS has confirmed that the figure files meet our requirements, please download the file via the download option, and include these NAAS processed figure files when submitting your revised manuscript.

**Reproducibility:**

To enhance the reproducibility of your results, we recommend that authors of applicable studies deposit laboratory protocols in protocols.io, where a protocol can be assigned its own identifier (DOI) such that it can be cited independently in the future. Additionally, PLOS ONE offers an option to publish peer-reviewed clinical study protocols. Read more information on sharing protocols at https://plos.org/protocols?utm_medium=editorial-email&utm_source=authorletters&utm_campaign=protocols

# Author comment: Author response to Decision Letter 1

**DOI:** 10.1371/journal.pgen.1012147.r002

16 Sep 2026

Attachment Submitted filename: Response to Reviewers 12-09-26.docx

# Peer review: Decision Letter 1

**DOI:** 10.1371/journal.pgen.1012147.r003

**Authors:** Gustavo Goldman, Benjamin Schwessinger

8 Oct 2026

PGENETICS-D-26-00432R1

The lipid raft marker flotillin FloA drives relocalization of the plasma membrane H^+^-ATPase PmaA as a protective response to calcium stress

PLOS Genetics

Dear Dr. Brakhage,

Thank you for submitting your manuscript to PLOS Genetics. After careful consideration, we feel that it has merit but does not fully meet PLOS Genetics's publication criteria as it currently stands. Therefore, we invite you to submit a revised version of the manuscript that addresses the points raised during the review process.

Please submit your revised manuscript within by Nov 07 2026 11:59PM. If you will need more time than this to complete your revisions, please reply to this message or contact the journal office at plosgenetics@plos.org. When you're ready to submit your revision, log on to https://www.editorialmanager.com/pgenetics/ and select the 'Submissions Needing Revision' folder to locate your manuscript file.

Please include the following items when submitting your revised manuscript:

* A letter that responds to each point raised by the editor and reviewer(s). You should upload this letter as a separate file labeled 'Response to Reviewers'. This file does not need to include responses to formatting updates and technical items listed in the 'Journal Requirements' section below.

* A marked-up copy of your manuscript that highlights changes made to the original version. You should upload this as a separate file labeled 'Revised Manuscript with Track Changes'.

* An unmarked version of your revised paper without tracked changes. You should upload this as a separate file labeled 'Manuscript'.

If you would like to make changes to your financial disclosure, competing interests statement, or data availability statement, please make these updates within the submission form at the time of resubmission. Guidelines for resubmitting your figure files are available below the reviewer comments at the end of this letter.

As the corresponding author, your ORCID iD is verified in the submission system and will appear in the published article. PLOS supports the use of ORCID, and we encourage all coauthors to register for an ORCID iD and use it as well. Please encourage your coauthors to verify their ORCID iD within the submission system before final acceptance, as unverified ORCID iDs will not appear in the published article. Only the individual author can complete the verification step; PLOS staff cannot verify ORCID iDs on behalf of authors.

We look forward to receiving your revised manuscript.

Kind regards,

Gustavo Henrique Goldman, Ph.D.

Guest Editor

PLOS Genetics

Benjamin Schwessinger

Section Editor

PLOS Genetics

Aimée Dudley

Editor-in-Chief

PLOS Genetics

Anne Goriely

Editor-in-Chief

PLOS Genetics

**Additional Editor Comments (if provided):**

Your manuscript has been reviewed by the same three initial reviewers. Two accepted the manuscript, while one requested minor revisions. Please send a revised version of the manuscript that addresses the first reviewer's comments and suggestions point by point.

**Reviewers' comments:**

Reviewer's Responses to Questions

**Comments to the Authors:**

**Please note here if the review is uploaded as an attachment.**

Reviewer #1: The authors made significant changes to the revised manuscript that have improved the support for their conclusions. However, there are still some concerns that need to be addressed.

1. Page 9, Figure 3. GO term analysis should be presented to show whether there was a statistically significant enrichment of endocytic proteins.

2. Table 1. Define what is meant by LQF intensities. What are the units for the values reported as the “mean”. Is it reported as log2? Or as raw counts?

3. Figure 4A. The label “raft” under the blots should be changed to DRM, since that is what was analyzed. As the authors pointed out, they are not always the same.

4, Line 173 states that 111 proteins were significantly enriched compared to the negative control. The authors should state what is the negative control in this case. Also, it was stated on line 172 that 270 proteins were uniquely detected in the floA-3xHA-miniTurboID strain. This is confusing.

5. Line 222. Pma1-cherry and FloA-GFP do not look punctate. They seem relatively evenly distributed in the membrane. The authors should present an airyscan image of the top of the cell to provide support for their conclusion that these proteins are in puncta.

6. Line 276. The authors state that “a substantial fraction of conidia failed to internalize PmaA-mCherry”. This should be quantified and reported as the percent of cells that failed to internalize PmaA-mCherry and it should also be stated how many independent experiments were carried out.

7. Figure S9. The sensitivity to oxypurinol provides some indirect evidence. However, it would have been more compelling to see if UapA-GFP or any other plasma membrane protein stayed on the membrane in the floA mutant treated with Calcium, or if it was internalized like Pma1.

8. Define the floA-C strain shown in Figure 6. I assume it is the complemented strain? This should be described in the Figure Legend.

9. Line 342 Define “SPFH family”

Minor comments:

-The term knockout always seems like lab jargon to me. It would be clearer to say deletion mutant or insertion mutant, as this better describes the type of “knockout” mutation. However, I leave it to the authors if they want to make this change or not.

-Line 130-131. The text states that there are three CrzA-binding motifs in Af floA, but Fig S6 shown only two motifs.

Reviewer #2: Dear authors, Thank you for providing your revised and improved ms. With that, i can recommend publication of the ms

Reviewer #3: This version of the manuscript is improved by the addition of new data on FloA. I do not have any further comments on the manuscript.

**********

**Have all data underlying the figures and results presented in the manuscript been provided?**

Large-scale datasets should be made available via a public repository as described in the *PLOS Genetics*  data availability policy, and numerical data that underlies graphs or summary statistics should be provided in spreadsheet form as supporting information.

Reviewer #1: Yes

Reviewer #2: Yes

Reviewer #3: Yes

**********

PLOS authors have the option to publish the peer review history of their article (what does this mean?). If published, this will include your full peer review and any attached files.

If you choose “no”, your identity will remain anonymous but your review may still be made public.

**Do you want your identity to be public for this peer review?**  For information about this choice, including consent withdrawal, please see our Privacy Policy.

Reviewer #1: No

Reviewer #2: No

Reviewer #3: **Yes:**  Praveen Juvvadi

[NOTE: If reviewer comments were submitted as an attachment file, they will be attached to this email and accessible via the submission site. Please log into your account, locate the manuscript record, and check for the action link "View Attachments". If this link does not appear, there are no attachment files.]

**Figure resubmission:**

While revising your submission, we strongly recommend that you use PLOS’s NAAS tool (https://ngplosjournals.pagemajik.ai/artanalysis) to test your figure files. NAAS can convert your figure files to the TIFF file type and meet basic requirements (such as print size, resolution), or provide you with a report on issues that do not meet our requirements and that NAAS cannot fix.

After uploading your figures to PLOS’s NAAS tool - https://ngplosjournals.pagemajik.ai/artanalysis, NAAS will process the files provided and display the results in the "Uploaded Files" section of the page as the processing is complete. If the uploaded figures meet our requirements (or NAAS is able to fix the files to meet our requirements), the figure will be marked as "fixed" above. If NAAS is unable to fix the files, a red "failed" label will appear above. When NAAS has confirmed that the figure files meet our requirements, please download the file via the download option, and include these NAAS processed figure files when submitting your revised manuscript.

**Reproducibility:**

To enhance the reproducibility of your results, we recommend that authors deposit laboratory protocols in protocols.io, where a protocol can be assigned its own identifier (DOI) such that it can be cited independently in the future. Additionally, PLOS ONE offers an option to publish peer-reviewed clinical study protocols. Read more information on sharing protocols at https://plos.org/protocols?utm_medium=editorial-email&utm_source=authorletters&utm_campaign=protocols

# Author comment: Author response to Decision Letter 2

**DOI:** 10.1371/journal.pgen.1012147.r004

16 Oct 2026

Attachment Submitted filename: Response to Reviewers 15-10-26.docx

# Editor report: Decision Letter 2

**DOI:** 10.1371/journal.pgen.1012147.r005

**Authors:** Gustavo Goldman, Benjamin Schwessinger

20 Oct 2026

Dear Dr Brakhage,

We are pleased to inform you that your manuscript entitled "The lipid raft marker flotillin FloA drives relocalization of the plasma membrane H^+^-ATPase PmaA as a protective response to calcium stress" has been editorially accepted for publication in PLOS Genetics. Congratulations!

Before your submission can be formally accepted and sent to production you will need to complete our formatting changes, which you will receive in a follow up email. Please be aware that it may take several days for you to receive this email; during this time no action is required by you. Please note: the accept date on your published article will reflect the date of this provisional acceptance, but your manuscript will not be scheduled for publication until the required changes have been made.

Once your paper is formally accepted, an uncorrected proof of your manuscript will be published online ahead of the final version, unless you’ve already opted out via the online submission form. If, for any reason, you do not want an earlier version of your manuscript published online or are unsure if you have already indicated as such, please let the journal staff know immediately at plosgenetics@plos.org.

In the meantime, please log into Editorial Manager at https://www.editorialmanager.com/pgenetics/, click the "Update My Information" link at the top of the page, and update your user information to ensure an efficient production and billing process. Note that PLOS requires an ORCID iD for all corresponding authors. Therefore, please ensure that you have an ORCID iD and that it is validated in Editorial Manager. To do this, go to ‘Update my Information’ (in the upper left-hand corner of the main menu), and click on the Fetch/Validate link next to the ORCID field.  This will take you to the ORCID site and allow you to create a new iD or authenticate a pre-existing iD in Editorial Manager.

If you have a press-related query, or would like to know about making your underlying data available (as you will be aware, this is required for publication), please see the end of this email. If your institution or institutions have a press office, please notify them about your upcoming article at this point, to enable them to help maximise its impact. Inform journal staff as soon as possible if you are preparing a press release for your article and need a publication date.

Thank you again for supporting open-access publishing; we are looking forward to publishing your work in PLOS Genetics!

Yours sincerely,

Gustavo Henrique Goldman, Ph.D.

Guest Editor

PLOS Genetics

Benjamin Schwessinger

Section Editor

PLOS Genetics

Aimée Dudley

Editor-in-Chief

PLOS Genetics

Anne Goriely

Editor-in-Chief

PLOS Genetics

www.plosgenetics.org

BlueSky: @plos.bsky.social

----------------------------------------------------

Comments from the reviewers (if applicable):

----------------------------------------------------

**Data Deposition**

If you have submitted a Research Article or Front Matter that has associated data that are not suitable for deposition in a subject-specific public repository (such as GenBank or ArrayExpress), one way to make that data available is to deposit it in the Dryad Digital Repository. As you may recall, we ask all authors to agree to make data available; this is one way to achieve that. A full list of recommended repositories can be found on our website.

The following link will take you to the Dryad record for your article, so you won't have to re‐enter its bibliographic information, and can upload your files directly:

http://datadryad.org/submit?journalID=pgenetics&manu=PGENETICS-D-26-00432R2

More information about depositing data in Dryad is available at http://www.datadryad.org/depositing. If you experience any difficulties in submitting your data, please contact help@datadryad.org for support.

Additionally, please be aware that our data availability policy requires that all numerical data underlying display items are included with the submission, and you will need to provide this before we can formally accept your manuscript, if not already present.

----------------------------------------------------

**Press Queries**

If you or your institution will be preparing press materials for this manuscript, or if you need to know your paper's publication date for media purposes, please inform the journal staff as soon as possible so that your submission can be scheduled accordingly. Your manuscript will remain under a strict press embargo until the publication date and time. This means an early version of your manuscript will not be published ahead of your final version. PLOS Genetics may also choose to issue a press release for your article. If there's anything the journal should know or you'd like more information, please get in touch via plosgenetics@plos.org.

# Editor report: Acceptance letter

**DOI:** 10.1371/journal.pgen.1012147.r006

**Authors:** Gustavo Goldman, Benjamin Schwessinger

PGENETICS-D-26-00432R2

The lipid raft marker flotillin FloA drives relocalization of the plasma membrane H^+^-ATPase PmaA as a protective response to calcium stress

Dear Dr Brakhage,

We are pleased to inform you that your manuscript entitled "The lipid raft marker flotillin FloA drives relocalization of the plasma membrane H^+^-ATPase PmaA as a protective response to calcium stress" has been formally accepted for publication in PLOS Genetics! Your manuscript is now with our production department and you will be notified of the publication date in due course.

The corresponding author will soon be receiving a typeset proof for review, to ensure errors have not been introduced during production. Please review the PDF proof of your manuscript carefully, as this is the last chance to correct any errors. Please note that major changes, or those which affect the scientific understanding of the work, will likely cause delays to the publication date of your manuscript.

Soon after your final files are uploaded, unless you have opted out or your manuscript is a front-matter piece, the early version of your manuscript will be published online. The date of the early version will be your article's publication date. The final article will be published to the same URL, and all versions of the paper will be accessible to readers.

For Research Articles, you will receive an invoice from PLOS for your publication fee after your manuscript has reached the completed accept phase. If you receive an email requesting payment before acceptance or for any other service, this may be a phishing scheme. Learn how to identify phishing emails and protect your accounts at https://explore.plos.org/phishing.

Thank you again for supporting PLOS Genetics and open-access publishing. We are looking forward to publishing your work!

With kind regards,

Janani Seenivasan

PLOS Genetics

On behalf of:

The PLOS Genetics Team

Carlyle House, Carlyle Road, Cambridge CB4 3DN | United Kingdom

plosgenetics@plos.org | +44 (0) 1223-442823

plosgenetics.org | Twitter: @PLOSGenetics
